# Influence of Tear Protein Deposition on the Oxygen Permeability of Soft Contact Lenses

**DOI:** 10.1155/2017/5131764

**Published:** 2017-02-09

**Authors:** Se Eun Lee, So Ra Kim, Mijung Park

**Affiliations:** Department of Optometry, Seoul National University of Science and Technology, Seoul 01811, Republic of Korea

## Abstract

*Purpose*. To investigate the effect of tear protein deposition on the change in oxygen permeability (*Dk*) of soft contact lenses (SCL).* Methods*. Three hydrogel lenses (polymacon, nelfilcon A, and etafilcon A) and two silicon hydrogel lenses (lotrafilcon A and balafilcon A) were investigated. Etafilcon A lenses were incubated in artificial tear solution for 1, 6, 12, and 48 h, whereas the other SCL were incubated for 1, 3, 7, and 14 days. Oxygen permeability was measured using the polarographic method, and lenses were stacked in four layers to correct the boundary effect.* Results*. The* Dk* of all investigated SCL was decreased by the protein deposition. Silicone hydrogel lenses showed a smaller deposition of artificial tear proteins than conventional hydrogel lenses. However, their* Dk* was reduced twofold than those of 3 conventional hydrogel lenses when compared at the same level of protein deposition. Despite a large amount of total deposited protein in etafilcon A lenses, their* Dk* was more stable than other SCL.* Conclusions*. From the results, it was revealed that the* Dk* of SCL is different from the value provided by manufacturers because of the tear protein deposition on surface and/or in pore of SCL; however, the degree of* Dk* change in SCL was not simply correlated with the amount of tear protein deposition. Thus, it is considered that the correlation between tear protein deposition and properties of lens materials affects* Dk* change.

## 1. Introduction

Soft contact lenses (SCL) used for visual correction and/or cosmetic reasons act as barriers against the oxygen transportation into the cornea, although the degree varies depending on the characteristic of lens materials, which ultimately affect the lens wearers' tear and corneal metabolism [1–3]. Thus, conventional hydrogel lenses with low oxygen permeability (*Dk*) may cause hypoxic condition of the cornea and can further lead to clinical issues such as corneal edema, corneal neovascularization, corneal acidosis, epithelial keratitis, and endothelial polymegethism [4]. Particularly, the change of corneal epithelium induced by contact lens wear is known to be found more often in Asian wearers than non-Asians [5]. Vice versa, the tear and corneal metabolism affected by SCL wear may also affect the lenses and cause changes in lens parameters such as water content and lens surface [6]. Therefore, new materials of SCL with higher* Dk* above 150 units [10^−11^ cm^2^/s (mL O_2_/mL × mmHg)] have been developed to reduce the hypoxic condition of the cornea [7, 8]. The* Dk* value provided by the manufacturers represents that of packaged SCL, applicable to the specific temperature, pH, osmolarity, and buffer solution. In our previous study, the surrounding environmental conditions such as pH, osmolality, and buffering system were shown to alter* Dk* value of SCL, and the changes differed according to the characteristic of lens polymers [9]. It was also reported that a large amount of artificial tear protein was deposited on only the ionic lenses with high water content, which could lead to a decrease in oxygen permeability of as much as 7 *Dk* units by reducing the amount of free water by 10% [10]. However, to the best of our knowledge, no studies have investigated oxygen transmission according to the interactions of SCL materials and the amount of protein deposition.

Generally, SCL materials are divided into four groups by the Food and Drug Administration (FDA) Guidance according to the water content and surface characteristics of lens materials, which influence not only comfort but also protein and lipid deposition on SCL [6]. Indeed, it has been reported that the amount of deposited protein on FDA group IV contact lenses is known to be 22 times and 17 times greater compared with that of FDA groups I and II contact lenses, respectively [11, 12]. Difference in tear protein deposition on SCL is caused by variations in factors such as the interaction between ionic proteins and the charge of the lens surface, the size of tear proteins, and the selective affinity of proteins in relation to surface wetting [13–15]. Thus, lysozymes and tear proteins are predominately deposited on ionic or high water containing SCL [16]. On the other hand, senofilcon A lens, a silicone hydrogel lens, has relatively low levels of lysozyme and lactoferrin deposition, and the levels of lysozyme, lactoferrin, and lipocalin-1 absorption in balafilcon A lens, another silicone hydrogel lens, have been shown to be statistically higher than those on senofilcon A lens [17]. As stated above, tear protein deposition can induce change in lens parameters such as total diameter, back optic zone radius, and water content of SCL as well as vision-related changes [10, 16, 18]. Therefore, we hypothesized that the* Dk* value of SCL after lens wear may be different by the characteristic of lens materials since tear protein deposition on SCL varies depending on their pertaining FDA groups. When wearing SCL, lenses are exposed to a changing tear film composition and structure induced by changes in lens parameters, environmental surroundings, and wearers' physiological and pathological conditions [19, 20]. Thus, in vivo study would be difficult to conduct in a controlled manner and further draw a conclusion from the results. In this study, an artificial tear solution consisting of three major tear proteins, lysozyme, globulin, and albumin was employed to absorb the various SCL in vitro. Therefore, this study aimed to assess whether the* Dk* change induced by protein deposition in SCL is influenced by the characteristic of lens materials beside the amount of deposited proteins.

## 2. Materials and Methods

### 2.1. Contact Lenses

Three hydroxyethyl methacrylate- (HEMA-) based contact lenses and two silicone hydrogel contact lenses were investigated in this study (Table 1). The back vertex power of all lenses investigated was −3.00*D*.

### 2.2. Incubation of Contact Lenses in Artificial Tear Solution

A single lens of each type of five SCL was incubated in an individual vial filled with 5 ml of artificial tear solution comprising 0.54 g/100 ml of bovine serum albumin (Amresco, USA), 0.18 g/100 ml of mucin (Sigma, USA), 0.13 g/100 ml of lysozyme (Lysozyme EGG White, Amresco, USA), and 0.001 g/100 ml of CaCl_2_ (Amresco, USA) in 0.01 M phosphate-buffered saline (PBS, pH 7.4) based on a previous study [21]. In total, 10 vials for each of the 5 SCL were prepared to measure oxygen permeability and protein deposition and were incubated on a shaker (CR300, FINEPCR, Korea) at 50 rpm, at room temperature. In a previous study, the amount of deposited protein on etafilcon A lenses was found to be significantly different compared with other contact lenses [16]. Thus, the FDA group IV lenses (etafilcon A) were incubated for 1, 6, 12, and 48 h, whereas the other lenses were incubated for 1, 3, 7, and 14 days to absorb the comparable range of artificial tear protein on SCL regardless of lens materials, since the present study aimed to investigate the effect of amount of protein deposition by various lens materials, not by incubation time, on* Dk* of SCL.

### 2.3. Measurements of Oxygen Permeability

After incubation with an artificial tear solution, SCL were rinsed with PBS (pH 7.4) in order to remove any unbound proteins on the lens surface. Then, the* Dk* of SCL was determined using the commonly utilized Createch permeometer (201T, Rehder Development Co., USA).* Dk* measurement was conducted in a temperature- and humidity-controlled box (Wisecube® WTH-E 155, Daihan Scientific, Korea) at 35°C and above 95% relative humidity according to the ISO standards [22, 23]. SCL were stacked in two, three, and four layers to measure the thicknesses (*t*) when their current reached a stable state, which is the same method used to correct a boundary effect in previous studies [24]. The test was repeated twice with different contact lenses. The* Dk*/*t* was calculated based on the current of SCL at a stable state. Origin Pro 8 software (OriginLab, USA) was used to draw a linear graph with *t*/*Dk* on the vertical axis and sample thickness on the horizontal axis. The slope of the line and the correlation coefficient was obtained though this linear graph. An electronic thickness gauge (ET-3 electronic thickness gauge, Rehder Development Co., USA) was used to measure single and multiple layers of contact lens samples. The range of* Dk* in this study varied between 8.4 and 140 units [10^−11 ^cm^2^/s (mL O_2_/mL × mmHg)]. To compare the differences induced by the amount of deposited proteins,* Dk* was normalized as relative* Dk*. The trend line and the equation were inserted through the correlation analysis of relative* Dk* and protein amount. Statistical significance was confirmed based on the FATT method stated in ISO 18369-2, 4 [22, 23].

(1) Relative  Dk=Dk′Dk,

where (*Dk*) is the* Dk* value of a contact lens before incubation in an artificial tear solution and (*Dk*)′ is the* Dk* value of a contact lens after incubation in an artificial tear solution.

### 2.4. Extractions and Assay of Deposited Proteins

After measuring* Dk* of SCL, the protein deposited in/on each lens was extracted with a sodium dodecyl sulfate (SDS) buffer (2% SDS and 0.1% dithiothreitol in 0.01 M Tris buffer) at 95°C for 15 min [25]. The extracted protein solutions were assayed for protein quantification by the Lowry method [26].

## 3. Results

### 3.1. Amount of Protein Deposition

Total protein deposition varied according to the lens materials (Table 2). In hydrogel lenses, the nonionic lenses, polymacon (FDA group I) and nelfilcon A (FDA group II), showed smaller total protein deposition compared with that of the ionic lens, etafilcon A. That is, total protein depositions measured were 9.7~26.1 *μ*g/lens in polymacon lenses and 13.8~57.4 *μ*g/lens in nelfilcon A lenses when incubated in an artificial tear solution for the period as stated above whereas etafilcon A lenses absorbed more protein than other lenses with a range of 770.3~2,589.4 *μ*g/lens in accordance with the incubation time from 1 h to 48 h. On the other hand, silicone hydrogel lenses, lotrafilcon A (FDA group I) and balafilcon A (FDA group III) showed the smallest protein deposition among the all tested SCL. Total protein depositions measured were 10.2~18.64 *μ*g/lens in lotrafilcon A and 6.07~12.7 *μ*g/lens in balafilcon A, respectively, in the same period as those for nelfilcon A lenses. Out of all lenses investigated, etafilcon A lenses exhibited the largest amount of deposited protein.

### 3.2. Relationship between Oxygen Permeability and Protein Deposition

**Figure 1:**
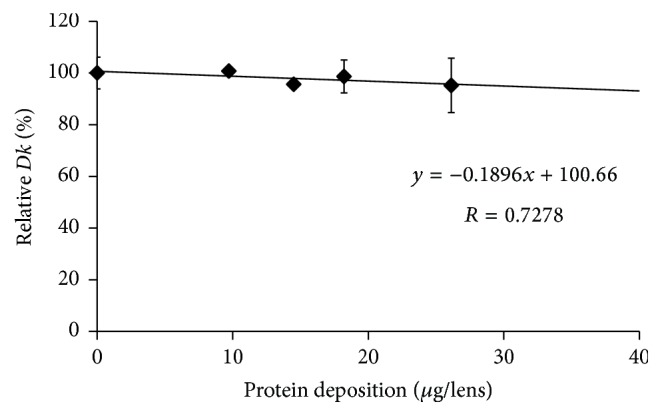
Relationship between relative* Dk* and amount of protein deposition on polymacon lens.

**Figure 2:**
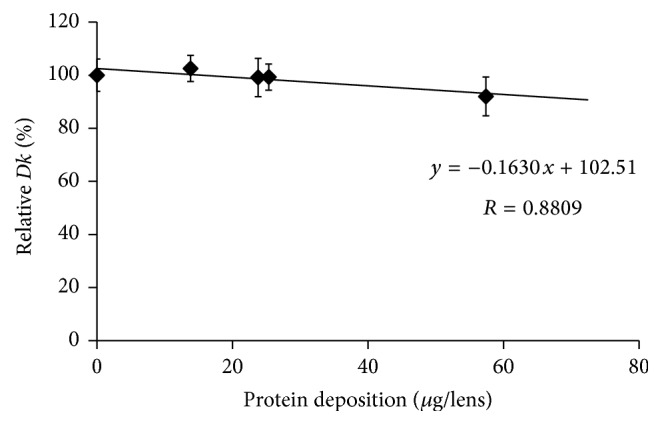
Relationship between relative* Dk* and amount of protein deposition on nelfilcon A lens.

**Figure 3:**
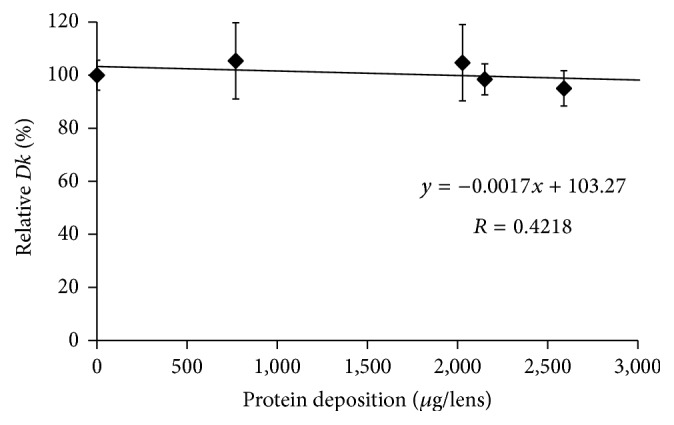
Relationship between relative* Dk* and amount of protein deposition on etafilcon A lens.

**Figure 4:**
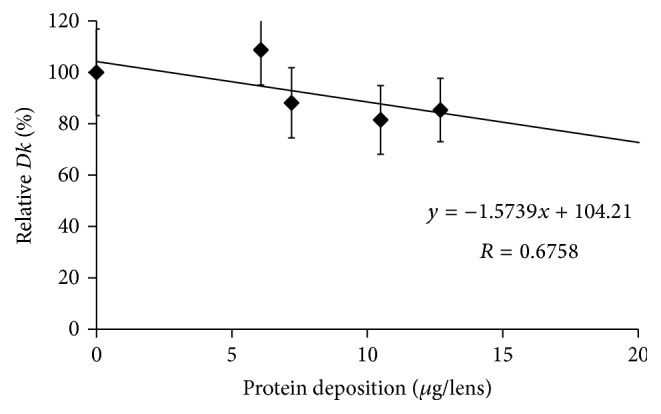
Relationship between relative* Dk* and amount of protein deposition on lotrafilcon A lens.

**Figure 5:**
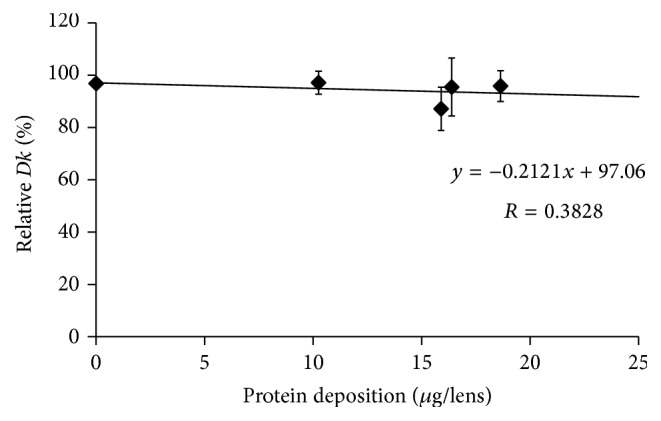
Relationship between relative* Dk* and amount of protein deposition on balafilcon A lens.

The* Dk* values of SCL before incubation in an artificial tear solution were compared with the values after incubation for 14 days, except for etafilcon A lenses. Figures 1, 2, 3, 4, and 5 show the differences in “relative* Dk*” between each lens type and the regression line of each lens. The* Dk* values of all SCL were reduced to varying extents by protein deposition on the tear film, depending on the lens material. The regression line of* Dk* showed a tendency to decrease when the amount of protein on the contact lenses increased. Indeed, relative* Dk* of polymacon and nelfilcon A lenses decreased by 4.77% and 7.99%, respectively, after the incubation for 14 days with an artificial tear solution compared with preincubation values. The relative* Dk* values of the silicone hydrogel lenses, lotrafilcon A and balafilcon A, decreased by 14.68% and 1.01%, respectively, after 14-day incubation. For etafilcon A lens, the relative* Dk* reduced by 4.98% of the baseline value after 48 h incubation. The* Dk* of silicone hydrogel lenses showed a greater decreasing trend than hydrogel lenses. Lotrafilcon A lenses exhibited the largest reduction in* Dk* among all investigated SCL even though its protein deposition was the smallest among the investigated SCL.

## 4. Discussion

Proteins were primarily deposited on the contact lenses from the tear fluid. The adhesion of proteins derived from tear substances on contact lenses is associated with diminished visual acuity, a feeling of dryness, and discomfort [27, 28]. Deposition depends on a number of factors including the protein charge and size, environmental pH, substrate charge and water content, and competition between the various tear film constituents [29, 30].

In the present study, the influence of deposited proteins on* Dk* of SCL was investigated. For this purpose, lenses were incubated in an artificial tear solution containing albumin, globulin, and lysozyme, and deposited proteins were assayed to investigate the relationship between the amount of protein deposition and the* Dk* of different contact lenses. The* Dk* of the SCL investigated varied between 8.4 and 140 units [10^−11 ^cm^2^/s (mL O_2_/mL × mmHg)]. We compared the relative change in* Dk* following different periods of incubation in artificial tear solution between SCL types. Based on the slope of each regression line of 5 different SCL (Figures 1–5), assuming that 50 *μ*g of tear protein is equally deposited in all lenses, the relative* Dk* of polymacon and nelfilcon A lenses will be 91.2% and 94.4%, respectively, compared with the value of preincubation with artificial tears. In the case of lotrafilcon A and balafilcon A, the relative* Dk* will be 25.5%, and 86.6%, respectively. On the other hand, etafilcon A lenses will not be affected by tear protein deposition of 50 *μ*g. Thus, the relative* Dk* of etafilcon A lenses was less affected by protein deposition, even though the total amount of deposited proteins increased up to 2,589.4 *μ*g/lens in the present study.

In conventional hydrogel lenses, oxygen is dissolved in water and transported through the materials to the cornea; therefore, the water content of lens materials is a key factor in oxygen transmissibility [31]. Mirejovsky et al. reported that a large amount of artificial tear protein on high water ionic lenses induced the reduction of free water content in the lenses, which led to a decrease in oxygen permeability when incubated for 14 days [10]. However, the protein deposition was not measured and analyzed based on the decrease in* Dk* of high water ionic lenses. In the present study, even though the amount of protein deposition on etafilcon A lenses was much higher than that of silicone hydrogel lenses, the decrease of* Dk* was not much larger. This can be explained by the possibility that the shorter incubation time might not be enough to change lens parameters such as free water content in etafilcon A lenses.

On the other hand, the relative* Dk* of lotrafilcon A lenses showed a greater reduction even though very little amounts of total deposited proteins were measured compared with conventional hydrogel lenses. In contrary to conventional hydrogel lenses, the high oxygen solubility in the silicone segment of the polymer causes an increase in permeability, and water-borne transport less affects oxygen transmissibility in silicone hydrogel lenses [32]. In a study by Compañ et al., lotrafilcon A and balafilcon A lenses were found to contain organosilicone moieties that enhanced the oxygen permeability in the water phase [33]. Indeed, water content in silicone hydrogel lenses tested was 24% for lotrafilcon A lenses and 36% for balafilcon A lenses though their* Dk* was high as 140 and 101 units [10^−11 ^cm^2^/s (mL O_2_/mL × mmHg)], respectively (Table 1). Therefore, the reduction of* Dk* in silicone hydrogel lenses might not be mainly caused by the change of free water content unlike conventional hydrogel lenses.

Among the silicone hydrogel materials, the amount of deposited proteins on lotrafilcon A lenses was less than balafilcon A lenses; however, the* Dk* value was substantially reduced compared with that of balafilcon A lenses. This is supposed to be related to differences in the surface properties of contact lenses. The surfaces of silicone hydrogel lenses are treated to reduce lipid deposition, improve the wettability of materials, and reduce the degree of deposition. Lotrafilcon A lenses are permanently modified in a gas plasma reactive chamber to create an ultrathin (25 nm) lens, a high refractive index, and a continuous hydrophilic surface. The surface of balafilcon A lenses are treated in a gas plasma reactive chamber, resulting in the silicone components on the lens surface becoming hydrophilic [34]. However, the surface of balafilcon A lenses is rougher than that of lotrafilcon A lenses [34]; thus, artificial tear proteins were more absorbed on balafilcon A lenses than lotrafilcon A lenses. Conversely, the* Dk* change was larger in lotrafilcon A lenses, which probably resulted from a change in the oxygen transport system inside lenses rather than surface changes due to protein deposition.

The studies on factors affecting the* Dk* of SCL have been continuously conducted since the decrease in* Dk* negatively affects the physiology of the cornea [9, 10, 24, 33]. Therefore, it is important to estimate the* Dk* during SCL wear because decreased* Dk* will cause side effects such as corneal edema, corneal striae, corneal folds, endothelial polymegethism, and corneal exhaustion syndrome [4, 6]. In the present study, SCL were hydrated in artificial tears and proteins were deposited, and then the degree of change in their* Dk* was evaluated according to the protein deposition. Protein deposition decreased* Dk* of SCL in a different manner probably by the changes in water content and oxygen transport pathway based on the characteristic of lens materials. However, the changes in lens parameters such as water content, wettability of the contact lenses, and radius of curvature were not measured in the present study; thus, it should be confirmed by further studies.

From the results, it was revealed that the degree of* Dk* reduction in SCL caused by tear protein deposition varied depending on the lens materials, which could be used in contact lens selection and the side effect prediction. However, future researches should be conducted to investigate the mechanism by which the specific tear protein decreases the* Dk* and the cause of* Dk* reduction by protein deposition depending on the characteristic of lens materials.

## 5. Conclusions

The present study was conducted to estimate the reduction of* Dk* induced by tear protein during SCL wear by measuring the change in* Dk* of 5 different SCL after incubation with an artificial tear solution consisting of lysozyme, albumin, and globulin. The results showed that there was a difference in the amount of protein deposition on each lens material, and the* Dk* of the lens decreased in all tested lenses due to protein deposition though the degree of* Dk* decrease was within the allowable error (number stated ±20%, ISO 18369). These results suggest that the changes in water content of lens materials, lens surface, and so on induced by SCL wear in vivo may change* Dk* values of the lenses claimed by the manufacturer depending on the characteristics of contact lens materials. To reduce the complication caused by reduced* Dk* of SCL during lens wear, further in vitro* and* in vivo studies should be conducted to figure out the mechanism of* Dk* change according to the physiological condition of the SCL wearers.

## Figures and Tables

**Table 1:** Characteristics of contact lenses.

Classification	Hydrogel			Silicone hydrogel	
Brand name	Optima FW	Focus dailies	1 day Acuvue	Focus night & day	Purevision

USAN^a^	Polymacon	Nelfilcon A	Etafilcon A	Lotrafilcon A	Balafilcon A

Claimed *Dk* (×10^−11^)^b^	8.4	26	21.4	140	101

Water content (%)	38.6	69	58	24	36

Polymer	pHEMA	PVA	pHEMA + MAA	DMA + TRIS + siloxane macromer	NVP + TPVC + NCVE + PBVC

Thickness at −3.00*D* (mm)	0.035	0.1	0.084	0.08	0.09

FDA Group	I	II	IV	I	III

PVA: polyvinyl alcohol; pHEMA: poly-2-hydroxyethyl methacrylate; MAA: methacrylic acid; DMA: N,N-dimethylacrylamide; TRIS: trimethylsiloxy silane; NVP: N-vinyl pyrrolidone; TPVC: tris-(trimethylsiloxysilyl)propylvinyl carbamate; NCVE: N-carboxyvinyl ester; PBVC: poly(dimethylsiloxy)di(silybutanol)bis(vinyl carbamate)). ^a^United States Adopted Name; ^b^Oxygen permeability value claimed by manufacturer; unit, (cm^2^/s) (mL O_2_/mL × mmHg).

**Table 2:** Amount of total protein deposition after incubation in artificial tear solution.

Lens materials		Incubation periods (days)				
		0	1	3	7	14
Polymacon	Protein deposition (*µ*g/lens)	0	9.7 ± 0.3	14.5 ± 1.3	18.2 ± 0.7	26.1 ± 1.4
Nelfilcon A			13.8 ± 1.8	25.3 ± 4.3	23.8 ± 2.2	57.4 ± 8.9
Lotrafilcon A			6.1 ± 0.3	7.2 ± 0.6	10.5 ± 0.9	12.7 ± 0.4
Balafilcon A			10.3 ± 0.5	15.9 ± 1.0	16.4 ± 1.7	18.6 ± 0.8

Lens materials		Incubation periods (h)				
		0	1	6	12	48

Etafilcon A	Protein deposition (*µ*g/lens)	0	770.3 ± 95.5	2026.9 ± 150.0	2150.6 ± 130.7	2589.4 ± 168.3
